# Research Methods in the Study of Intersectionality in Psychology: Examples Informed by a Decade of Collaborative Work With Majority World Women’s Grassroots Activism

**DOI:** 10.3389/fpsyg.2020.494309

**Published:** 2020-10-29

**Authors:** Shelly Grabe

**Affiliations:** Department of Psychology, University of California, Santa Cruz, Santa Cruz, CA, United States

**Keywords:** intersectionality, majority world, transnational feminism, women’s human rights, power

## Abstract

Very few theories have generated the kind of interdisciplinary and international engagement that marks the intellectual history of intersectionality, leaving some authors to suggest that intersectionality is the most important theoretical contribution that the field of women’s studies has made thus far. Yet, consideration of intersectionality as a research paradigm has yet to gain a wide foothold in mainstream psychology. The current article uses a program of multimethod research designed in partnership with, and intending to center the intersectional experiences of, majority world women to propose a research agenda for the empirical study of intersectionality. Specifically, it is suggested that a research agenda rooted in intersectional understandings requires that: (1) researchers think carefully about social categories of analysis and how their methodological choices can best answer those questions, (2) psychologists reposition their research questions to examine processes by which structural inequities lead to power imbalances and gender-based norms that sustain women’s experience of marginalization and oppression, and (3) we understand how intersectional experiences can be applied toward change. Intersectional investigations hold a key to interrupting the structural dimensions of power that result in egregious consequences to peoples’ social, economic, and political lives, but only if we radically restructure what we think about knowledge, our roles, and the products of our research.

## Introduction

Very few theories have generated the kind of interdisciplinary and international engagement that characterizes the intellectual history of intersectionality, leaving some authors to suggest that intersectionality is the most important theoretical contribution that the field of women’s studies has made thus far (McCall, 2005; Carbado et al., 2013). Yet, consideration of intersectionality as a research paradigm has yet to gain a wide foothold in mainstream psychology (Hancock, 2007). More than a decade ago, Elizabeth Cole (2009) put forward ideas suggesting we were on the verge of a tipping point by noting that psychologists were becoming increasingly concerned with, “the effects of race/ethnicity, gender, social class, and sexuality on outcomes such as health and well-being, personal and social identities, and political views and participation” (p. 170). Since that time, the topic has gained increasing momentum: a keyword search on “intersectionality” in PsycInfo at the time of this writing yielded 2,333 results, 92% of which were published in 2009 or later. Despite this growing interest, only slightly more than half of the publications appear in scholarly peer-reviewed journals. The lack of congruence between intellectual interest in intersectionality, and empirical support for related processes or experiences, may reflect that the promise of intersectionality has had slow uptake in psychology due to perceived methodological challenges (Bowleg, 2008; Shields, 2008). In this article I review a decade of work from my own program of multimethod research to illustrate and discuss how research methods and empirical findings from one particular program of research might inform an agenda for the empirical study of intersectionality in psychology.

## Intersectionality and Psychology

Critical race theorist Crenshaw (1989) is credited with introducing the now widely used term “intersectionality” to describe the analytic approach to understanding lived experience from the lens of multiple intersecting categories of oppression (Cole, 2009). A Google Scholar search lands over 110,000 hits on the topic and a number of books focused on intersectionality are now in circulation (e.g., Hankivsky et al., 2009; Collins and Bilge, 2016; Hancock, 2016). Crediting the emergence of this focus requires recognizing the rich history of intellectual contributions from women of Color that undoubtedly influenced the wide embrace of this concept. Crenshaw (1989, 1990) work on intersectionality holds a pioneering place in scholarship and activism because it exposed the failure of the legal system to handle more than one aspect of African American women’s marginalization (Hurtado, 2018). Although Crenshaw’s work did not explicitly examine additional social locations that also oppress women, her work provided a conceptual framework for considering multiple sources of oppression in analyzing women’s experience. The groundswell in this area of theorizing may be explained by the fact that other feminist scholars prior to Crenshaw (e.g., Combahee River Collective, 1977; Moraga and Anzaldua, 1983; Hooks, 1984; Hurtado, 1989; Collins, 1990) were writing influentially about intersectionality, even before the term was coined, by drawing attention to the limitations of centering investigations on only one aspect of women’s identities (e.g., gender or race). Moreover, although much of the writing on this topic emerged from Black feminist thought (e.g., Hooks, 1984; Collins, 1989), Chicana scholars also were identifying interlocking oppressions as a way to understand variations in the experiences among women (e.g., Anzaldúa, 1987; Hurtado, 1989). By now, a general consensus exists surrounding the definition of intersectionality. “The term intersectionality references the critical insight that race, class, gender, sexuality, ethnicity, nation, ability, and age operate not as unitary, mutually exclusive entities, but as reciprocally constructing phenomena that in turn shape complex social inequalities” (Collins, 2015, p. 2).

More than a decade ago several authors proposed that methodological challenges may have stymied scholars in psychology from adopting an intersectional approach with the same vigor that was present in other disciplines (Bowleg, 2008; Shields, 2008). Since that time, intersectionality has become an approach that is widely accepted and increasingly applied to research paradigms and questions within feminist psychology (Ceballo et al., 2015; Bowleg and Bauer, 2016; Else-Quest and Hyde, 2016; Rice and Grabe, 2019). Recently, a comprehensive in-depth special section devoted to intersectionality research and feminist psychology was published in *Psychology of Women Quarterly* with key articles exploring the use of intersectionality in both quantitative research and qualitative research (Del Toro and Yoshikawa, 2016). Yet, the current Special Issue in *Developmental Psychology* notes that an empirical basis from which to articulate intersectionality’s theoretical and practical relevance for identity development is still lacking (Azmitia and Mansfield, this issue). As the concept of intersectionality is gaining more concern and attention within psychology, finely tuned investigations will be necessary to avoid the concept becoming an empty construct (Harris and Patton, 2019).

In this article, I use investigations I conducted with community-based partners from the majority world^1^ as a guide to discussing methods and analyses that can prioritize the perspective of intersectionality. In over a decade worth of community-based work with women from Nicaragua and Tanzania, I used multiple, and sometimes mixed, methods that allowed me to interrupt standard approaches from within psychology. In doing so, I was able to develop a framework for investigating multiple sources of oppression in women’s lives. Although I did not set out with the specific intention to develop a program of research rooted in intersectionality, it was precisely because I entered my research partnerships with no formal training or agenda, that the program of research that developed (and will be described in this article) was open to be radically guided and influenced by activist women. The result led to partnerships, research questions, and samples that interrogated social identities and processes of power. Because the research partnerships that occurred in this program of research involved individuals with multiple social identities and locations, it required us to discuss how sociohistorical systems of influence led to identity-specific experiences and how those may influence our research projects. For me, I needed to acknowledge that I entered these research partnerships as a white, middle-class, monolingual, highly educated woman from the United States. Because my first project was in Nicaragua, it was critical to recognize that the United States has a long history of economic exploitation and armed conflict in Nicaragua, and that my government was deeply implicated in many of the repressive experiences of the women I was to work with. At the outset of all of my projects, I attempted to engage the differing social locations between me and the research partners by designing the studies with Mohanty (2003) suggestion that understanding women’s struggles for justice must involve illuminating “third world”^2^ women’s engagement with resistance to oppressive regimes. In the first investigation I conducted, this meant, for example, that the women identified the topic – the development of a social identity that emerged from resistance (e.g., “I moved from an object to a subject”) – and I, in turn, designed and administered an investigation that could examine the process by which they did so.

I have since collected and disseminated data based on large-scale mixed-methods investigations conducted in partnership with women in Nicaragua and Tanzania that document how processes of power impact women’s human rights (Grabe and Arenas, 2009; Grabe, 2010, 2012, 2015; Grabe et al., 2014, 2015; Grose and Grabe, 2014; Grabe and Dutt, 2015, 2019; Dutt et al., 2016; Dutt and Grabe, 2017, 2019). Findings from this research were used by the research partners to support and bolster their community involvements, and by me to put forward models in psychology for understanding transformative change (see Grabe, 2010, 2012 for detailed discussions of the collaboration and findings). On two occasions I have also presented the findings from the communities in which I worked at the United Nations (Grabe, 2014, 2019). The meeting ground for the women and me in these projects was the mutual awareness that there was a demand for the dissemination of feminist liberatory knowledge in the context of the political subjugation of women’s voices.

What follows in the remainder of the article is a review of that program of research and how it might inform future work that aims to take an intersectional approach. The review is organized into the four areas of inquiry put forward by the editors of the current Special Issue that are meant to help inform approaches to intersectional research: (1) who is included in a social identity category, (2) what roles to inequality and oppression play in the construction of intersectionality, (3) is there common ground for individuals who experience different intersectional configurations, and (4) with which methods is intersectionality best studied?

### Who Is Included in a Social Identity Category?

Despite the inclusive nature of intersectionality to capture processes related to power and subordination, many initial investigations into intersectionality within psychology have reflected a largely Western bias. More specifically, when considering the experience of gender subordination, most work in psychology has developed theories and understandings of gender oppression in Western, Educated, Industrialized, Rich, and Democratic (or WEIRD, see Henrich et al., 2010) contexts and imposed those perspectives across varied settings for understanding what has been conceptualized as “universal” gendered oppression, thereby questioning what we mean by the identity category “woman” (Kurtiş and Adams, 2015). Moreover, much of mainstream feminist psychology has employed methods that involve sampling predominately white undergraduate college students enrolled in psychology courses at United States universities (Marecek, 2012). These approaches to understanding gendered injustice are problematic not only because they develop understandings that may not be applicable across varied contexts, but also because they tend to treat women in majority world settings as powerless thereby serving to legitimize structures of domination (Kagitcibasi, 2002; Kurtiş and Adams, 2015; Grabe, 2016a).

In Hurtado (2018) work linking social identity theory (Tajfel, 1981) to intersectionality, she defines intersectionality as a “constellation of social identities that are the primary basis for power distribution *and* for stigmatization and subordination” (p. 162). She uses the term *intersectional identity* to refer to social identities that stem from being part of social categories and groups that result in specific political, social, and economic consequences, thereby allowing for the investigation of identity in a manner that prioritizes the systemic and structural dimensions of social life. For example, transnational and decolonial feminist scholars suggest that women’s experience in the majority world is inextricably linked to the systemic inequities of global power (e.g., colonialism, globalization; Sen and Grown, 1987; Narayan, 1997; Lugones, 2010; Bose, 2012; Grabe et al., 2015). Therefore, taking a critical view of marginalization to understand within group variability in social identity categories related to gender would require that psychologists expand their investigations to include women from the majority world who are confronting gendered injustices that are reproduced or exacerbated in local and global processes.

By broadening social identity categories to include the perspective of women whose experiences were related to systemic inequities of global power, I was positioned to ask and answer questions that had not yet been posed before in psychology; such as, how institutionalized gender-based inequalities grant men disproportionate power in society and thereby result in male control and dominance in a number of other areas, including interpersonal relationships. To privilege this perspective, the research conducted in both of the countries in which I worked was generated with a critical communicative methodology, whereby an egalitarian dialogue between the researcher (me) and the leaders of the community-based organizations was viewed as central to conducting the research (Gómez et al., 2010). One goal of collaborative community-based research, in this sense, was to challenge assumptions about knowledge production and raise questions about the purpose of research. The women working in the organizations knew experientially what scholars in academia had theorized: in order to reduce women’s susceptibility to violence in a complex, interconnected, and globalized world, interventions (and thereby investigations) should reflect multiple layers of society that demarcate women’s subordinate position (Heise, 1998; Grabe et al., 2015). This underscores that when establishing research samples and questions, attention should be given to whose voices are privileged in the production of scholarship, attempting to create a space for the expression of subjugated knowledge (Fals Borda, 1985). Within community-based psychological research, too often “partnerships” have been characterized by instrumentalist arrangements whereby participants are positioned as extractable data sources, rather than partners in collective efforts toward social change (Nelson et al., 2001). A partnership based on a shared interest in intersectionality needs to be rooted in common goals, which may not be primarily academic ones, but ones that have relevance to the community in which you are working.

#### The Community-Based Partners

The first organization I partnered with was Xochilt Acalt in Nicaragua. During a week of informal conversations, lead organizers explained that when women became land owners in a rural setting it served to restructure gendered power relations because it altered relationship dynamics and allowed women greater decision-making surrounding their bodies. Because customary practices the world over still largely prohibited women from owning land, Xochilt Acalt facilitated women’s landownership as part of mobilized efforts at shifting women’s status and social location. Years later, I was introduced to the director of a women’s grassroots organization in Tanzania that was employing precisely the same intervention: facilitating women’s landownership to transform the structural conditions in which women lived their lives. Although the social and political contexts in Nicaragua and Tanzania vary, land privatization issues in both countries were highly contested and each country has a shared history of women’s activism surrounding land reform^3^.

Both organizational settings in this program of research, Xochilt Acalt^4^ in Nicaragua, and the Maasai Women Development Organization (MWEDO)^5^ in Tanzania, used community mobilization to educate, serve, and advocate for women’s health and human rights by challenging gendered forms of structural inequities (the text describing these organizations is taken from Grabe et al., 2014). Within the broader global women’s movement, both organizations are revolutionary in that they are mobilized as agents of their own liberation and resist imposed international agendas that view women as recipients of service, rather than agents of change. Both organizations also involve participating members in political educational activities (e.g., literacy training, human rights workshops) that support women in challenging the systemic and structural dimensions of their social lives and to create solidarity relationships among women that may enhance their ability to engage in transformative action.

Data from participating members in each organization were collected as part of a large program of research combining quantitative and qualitative data to examine links between landownership and women’s experience of partner violence. As described elsewhere (Grabe et al., 2015), I quantitatively examined the links between landownership, relationship power, and women’s experience of partner violence. Multiple levels of analysis were used to decenter any one aspect as primary (e.g., land and relationship dynamics) and focused instead on the processes linking power to violence. I included a qualitative component to extend the analysis beyond the numbers; in other words, to examine how the social context and actual lived experience of women could help to better understand the role of landownership in reducing violence against women (Marecek, 2012). Integrating the qualitative component with quantitative approaches aligns with feminist principles that value the privileging of voices that have otherwise been silenced and allows us to see what happens when we diversity social identity categories in research (Stewart and Cole, 2007).

#### The Research Projects

To begin this research, I conducted focus groups with participating members of Xochilt Acalt in Nicaragua. The focus groups were centered on better understanding the role of land in creating gender norms and how *women’s* ownership of land could thereby alter the context of relationship power and reduce experiences of violence from partners. Following these initial discussions, I partnered with Xochilt Acalt and hired and trained a research team to administer a large scale survey that could empirically examine how multiple structures of power – in this case landownership and relationship power – related to women’s experience of violence. We administered household surveys to two different groups of women in a quasi-experimental manner – one group predominantly landowners and the other predominantly non-landowners. The two groups were chosen from the same geographical location within the country to closely match them on a number of economic, social, and cultural variables. To construct the intervention group, 174 women were randomly selected from a list of 380 women who had received assistance from Xochilt Acalt in facilitation of land titling. To construct the control group, 35 women each from five surrounding communities in the same municipality were randomly selected to participate. The total sample size in Nicaragua was 267 women (121 landowners and 146 non-landowners).

The study design in Tanzania began with a similar partnership between MWEDO and me. Discussions began with the organization’s director about the role of land in human rights issues in Maasailand, in general, and in processes that reduce violence among Maasai women, specifically. I shared the findings from the Nicaragua investigation with her and potential similarities and differences in the processes women experienced in each country were discussed. The survey from Nicaragua was used as a starting point, and I hired and trained a local team of Maasai women to establish which, if any, measures were culturally appropriate for Maasai women. To allow for a cross-country comparison, we retained a small but informative selection of scales that I also used in Nicaragua. We administered household surveys to three different groups of women: one that had received land titling and empowerment interventions, one that had received only empowerment interventions, and one that had not received either intervention to date. The groups of women were chosen from the same districts to ensure that they were culturally and geographically similar. To construct the intervention group we used a list of 71 women enrolled with MWEDO for the land and empowerment interventions. Of those women, 54 were administered the survey. The second group of women was selected from a list of 150 women in neighboring communities that had received empowerment interventions. We administered the survey to 114 of these women. To construct the third group, we used a list of women from a neighboring community that were slated to begin receiving interventions in the next year and we administered the survey to 59 women in this community. To make a cross-country analysis possible, we collapsed across the three groups of women to compare the landowners (*n* = 74) to the non-landowners (*n* = 151). The total sample size in Tanzania was 225 women. In both countries the survey questionnaires were developed in partnership with a research team, translated into Spanish and Swahili (respectively) by a member of the team, and then back-translated with a local speaker to ensure the meanings were properly conveyed before the surveys were piloted.

Qualitative interviews were also included at each site to gain a deeper understanding of how owning land was related to life experiences for women in each community. Following guidelines for qualitative interviewing, twenty women in each country were targeted for semi-structured interviews (Francis et al., 2010). Comparable numbers of women from each condition within each country were selected with the help of the local research team to reflect the age diversity and location in which they lived. Once saturation was achieved the interviews ceased (Francis et al., 2010). Nineteen women were interviewed in Nicaragua and 14 in Tanzania. Half of those interviewed in Nicaragua and one-third of those interviewed in Tanzania were landowners. Women were asked questions designed to assess their experiences in general (i.e., “Can you tell me a little about yourself? How do you normally spend your days?”) and to gain a better understanding of how landownership may affect women’s lives more specifically (e.g., “What do you think of women owning land?”). I conducted all of the interviews in women’s homes with the aid of an interpreter who was knowledgeable about the research methods and goals of the study.

Expanding the social category of “women” to include majority-world women, allowed for the first study published in this line of research to provide a theoretical framework for, and an examination of, hypotheses surrounding the role of landownership in shifting gender relations and women’s experience of partner violence that had been posed in the literature, but never empirically tested (Grabe, 2010). The quantitative surveys administered to women in rural Nicaragua revealed that landownership and participation with Xochilt Acalt were related to less traditional gender role ideology among women, suggesting that shifting the status and power of women through landownership resulted in more progressive ideas about gender among women. More progressive gender ideology was, in turn, a significant predictor of greater relationship power for women and receipt of less partner control. These findings are important because relationship power and partner control are known to influence women’s risk of violence (Jewkes, 2002), and indeed predicted women’s receipt of partner violence in this study. Specifically, we found that higher levels of women’s relationship power predicted less physical and sexual violence from their partners, whereas higher levels of partner control predicted greater incidence of psychological and sexual violence^6^. This study put psychology at the crossroads of women’s human rights, globalization, and social change by putting forth a model from the majority world for understanding how more than one structure of domination were linked in a manner that has dire consequences for women.

To replicate and extend these findings, I later published a mixed-method, cross country comparative paper that included 492 women from Nicaragua and Tanzania. Together with graduate student mentees, we used data from the quantitative surveys in both countries to produce structural equation models that demonstrated a robust parallel process in each country whereby women’s landownership predicted less physical and psychological violence from male partners because landownership enhanced relationship power among women in both locations (Grabe et al., 2015). The overall findings in this comparison suggest that the process by which women’s landownership relates to lower levels of physical and psychological violence operates similarly in each country. Promotion of these parallel findings is not rooted in the notion that women have universal experiences; rather it is rooted in a shared criticism of policies and societal practices that create structural conditions that limit women’s rights in their respective communities and locations. In this same paper, we used qualitative interviews to employ a thematic analysis used to identify patterns that repeatedly occurred within the interviews (Braun and Clarke, 2006). We identified and reported on two themes across the countries – one that fleshed out the transformative potential of land and another that illustrated how property ownership interrupted sociocultural structures of male power by strengthening women’s ability to address their needs independent of their husbands (see Grabe et al., 2015 for detailed findings and implications).

This line of research has demonstrated, through both quantitative surveys and qualitative interviews, that in order to address violence against women, men’s institutional power over resources (e.g., land) and interpersonal power over women need to be addressed simultaneously. By taking an approach to investigate how the patterned relations between women and men develop to predict domestic violence, we examined the dynamic interplay between different structures of domination as they occur at macro levels. Because ownership of land among women can substantially enhance their social status, it was related to women’s power and control within their relationships and with reduced levels of violence, regardless of varying cultural contexts. Centering majority world women’s perspective (i.e., diversifying social identity categories) through a multi method approach earned this cross-country research the Georgia Babladeis Best Paper award from the *Psychology of Women Quarterly* for its combination of critical theory, quasi-experimental design, and qualitative data that established casual structural influences on threats to women’s rights.

Demonstrating links between landowning and women’s power in two different regions of the majority world has implications for a proposed research agenda prioritizing intersectionality. In particular, researchers should think carefully about who is included in a social category, how this will influence the knowledge gained, and how the research design and assessments will prioritize whether and/or how within group variability of social identities may be captured. As Elizabeth Cole stated (2009), “considering groups that have traditionally been overlooked may lead researchers to hypothesize about different predictors” (p. 173). Therefore, because the challenges in intersectional research may not lie so much in the methodological plan, but on the research questions and samples, I implore researchers to consider working with communities, and still more, with communities that can flesh out the “complexities” that are relevant to the research questions.

### What Roles Do Inequality and Oppression Play in the Construction of Intersectionality?

Because a main tenet of intersectionality involves critique of how systems of power exacerbate or sustain oppression, a focus on social structures and systems of power in understanding lived experience and social justice is crucial (Collins, 1990). Although identifying structural patterns of inequality has long been the task of political and social theorists, liberation psychologist Martín-Baró (1994) argued that psychologists can and should reframe standard methods to consider that the root causes of oppression lie in both the structures *and* ideologies that underlie inequity (Grabe, 2016b,c). Decades later, Cole (2009) noted in much of the investigation of social categories, social and material inequality are often treated only implicitly despite that these categories reflect underlying historical and continuing political, material, and social inequity. Cole stated that, “Asking what role inequality plays draws attention to the ways that multiple category memberships position individuals and groups in asymmetrical relation to one another, affecting their perceptions, experiences, and outcomes” (p. 173). Indeed, focusing on individual-level investigations when attempting to understand intersectional experiences decontextualizes people from their political and social worlds and renders unexamined the structures of patriarchy, racism, classism, and capitalism that create conditions of risk and vulnerability (Fine, 1989). Nevertheless, to date, the bulk of mainstream psychology has studied women in micro-level investigations that separate them from their social contexts (Cortina et al., 2012).

Furthermore, simply acknowledging that multiple social locations intersect in a manner that may uniquely impact women’s lived experience is not sufficient to understand how to apply that knowledge in the course of conducting research (Shields, 2008). For example, as I have written elsewhere (Grabe, 2016a), there have been decades of feminist calls to put the question of gender differences aside to more closely examine how developmental *processes* involved in the psychological phenomenon surrounding gender may impact women’s experience of subordination (Hare-Mustin and Marecek, 1994; Marecek, 1995). Nevertheless, the more common approach to gender research in psychology has been to conduct group comparison tests to examine differences (or similarities) between women and men (Hyde, 2014). As important as findings from this approach have been in establishing broad inequities, the gender differences approach also inadvertently purports an essentialist model of gender that suggests that women, as a group, have universally shared experiences, relative to men as a group. And while gross inequities based on gender are widely documented, a focus on this approach overlooks differences between women and the contexts in which they live. The prevailing focus with the differences paradigm is reflected in a PSYCH Info search which revealed 89,343 articles in peer reviewed journals using the key word “gender differences.” In contrast, the key word “gender inequality” revealed only 4,008 articles with even fewer, 1,574, when using the key word “intersectionality.” Continuing to prioritize a gender differences approach will do little to further our understanding of processes by which inequality operates as a *system* of oppression at institutional, interpersonal, and intrapersonal levels (Shields, 2008). Research in psychology can be transformed by adopting an intersectional framework that understands gender *inequality* in the context of multiple levels of oppression.

#### Women’s Political Participation, as an Example

To illustrate examples that examine the role of inequality and oppression in the construction of intersectional experiences, I will review investigations I conducted on women’s political participation in the communities in which I worked. Globally, limited opportunities for women’s political participation and decision-making reflect a widespread societal problem substantiated and perpetuated through gender inequities, in particular, that operate at numerous levels of society. Although most strategies for women’s inclusion in politics attempt to promote the full and equal involvement of women, many attempt this without considering the larger social structures or psychosocial processes that may lead to transformative levels of political participation and decision-making among women (Mayoux, 1995; White, 1996). Moreover, most of the research and scholarship on women’s political participation exists in the disciplines of development and politics, with a near absence of investigating how the power structures in women’s lives prohibit their meaningful participation. In many places throughout the world, opportunities for women to participate politically are limited both by male power and women’s restricted access to resources, leaving women marginalized from participating as public decision-makers (Biglia, 2006). When considering women’s ability to participate in political spaces, several scholars have suggested that relationships rooted in power are directly related to women’s participation and inequality (Hassim, 1999; Sánchez and Martín-Sevillano, 2006).

In my program of research with community partners, I tested models that examined how the dynamics of structure, power, and agency enable (or limit) women’s political participation, specifically. This approach is compatible with Freire (1970) understanding of liberation, in which he argues that individuals are most likely to change their own circumstances by simultaneously working to challenge the social structures (e.g., gender inequity) that disadvantage them (Moane, 2003; Brodsky et al., 2012). Together with the community partners in Nicaragua and Tanzania, we designed a series of studies intending to capture: (1) the role of community-based interventions that address societal gender inequities, (2) the psychosocial and developmental processes that lead to meaningful changes in participation as women gain power and increase levels of decision-making in their communities, and (3) citizen participation outcomes that reflect engagement and decision-making among women (Grabe and Dutt, 2019). Because we were inherently interested in how macro-level influences (e.g., landownership, community intervention) interacted with interpersonal power dynamics to predict women’s political participation, I designed a large-scale survey that would allow us to empirically examine related psychosocial processes. And to better understand the social reality of individuals going through these processes, semi-structured interviews were also conducted in each location (again, Nicaragua and Tanzania).

#### Investigation and Findings

In the first study published in this line of work we examined how women’s landownership influenced the dynamics of relationship power and individual agency that enabled political participation among Maasai women in Tanzania (Grabe, 2015). This study was important because we measured a form of participation that does not fit a Western model of democratic representation and structure, and is therefore understudied. In Maasai communities of Tanzania formal decision-making occurs in meetings referred to by Tanzanian Maasai as *enkiguena.* These meetings are a formal institution for communication and decision-making in which all members of the community can participate in equally and are central to Maasai governing (Goldman and Milliary, 2014). Despite that all members of the community may participate equally in theory, women traditionally did not attend these meetings. Moreover, although everyone at an *enkiguena* has the freedom to speak and to be listened to, Maasai notions of respect and fear associated with social norms can inhibit some (e.g., women) from speaking (Hodgson, 2011). We hypothesized that women’s landownership, because it shifts structural power, would have the potential to address gendered obstacles to participation by impacting both women’s relational power and individual agency in a manner that would increase the likelihood of women speaking at *enkiguenas.*

Quantitative surveys designed to test these hypotheses revealed that landowning women reported experiencing significantly less difficulty with male partners prohibiting or controlling their ability to carry out everyday activities, than did non-landowning women. This is important because partner control was directly related to whether women were uncomfortable speaking at public meetings. Partner control also reduced women’s role in household decision-making. Whether women had comfort speaking at public meetings or engaged in more household decision-making, in turn, predicted their political participation. Specifically, when women reported discomfort speaking at meetings they, not surprisingly, were less likely to do so, reflecting lower participation and involvement in public decision-making. In contrast, when women exercised greater household decision making (which, remember was predicted by *lower* levels of partner control), they reported a *greater* likelihood of speaking at public meetings. These results provide support for the idea that it is not possible to adequately address women’s active participation in political spaces without simultaneously addressing the reality of inequality and oppression women confront as a result of the structural or relational circumstances of their lives. In other words, women’s political participation reflects an experience predicted by inequality and oppression at multiple levels (i.e., material and relational).

A similar study I conducted in Nicaragua investigated links between involvement in a community-based civic engagement program and women’s political participation (Grabe and Dutt, 2019). In this study, the focus was on examining how community-level resistance among women who have been excluded from political spaces has the potential to result in women’s meaningful participation in community decision-making. This investigation focused on assessing the community intervention which was aimed at challenging traditional gender ideology, thereby enhancing women’s autonomy and political efficacy, and increasing civic engagement and community leadership. Together with a graduate student, we hypothesized that participation in a community-driven intervention would shift traditional notions of gender ideology, thereby facilitating greater levels of agency and political efficacy among women, both of which we expected to relate to meaningful levels of women’s civic engagement and community leadership. To test these ideas, we conducted a second large scale multi method data collection conducted in partnership with Xochilt Acalt. Using survey data from 261 women, we found that women’s participation in a community intervention was directly related to developing a more progressive gender ideology, higher levels of agency, and higher levels of political efficacy. These findings lend support to Freire (1970) idea that collective organizing and raising awareness of one’s own social reality may need to take place before individuals can be empowered. Ideology, agency, and political efficacy, in turn, all predicted women’s political participation providing evidence that power is not only a political issue, but a psychosocial one. Three indicators of psychosocial empowerment: ideology, agency, and efficacy, all predicted whether or not women held a community leadership position. Women’s sense of political efficacy also predicted higher levels of civic engagement.

The findings from this last study were important because although issues of community intervention, citizen participation, and liberation are a large focus in community psychology, investigations that center women’s experiences are notably underexplored (Bond and Mulvey, 2000). This is well illustrated in a review that reported a lack of feminist analyses and scholarship related to women’s concerns in the community psychology literature (Angelique and Culley, 2000). It was reported in the review that between 1973 and 1997, of the 2,178 articles published in the *American Journal of Community Psychology* and the *Journal of Community Psychology*, only 9.8% were considered women relevant and only 3% were considered feminist. Of those that centered women’s experience, mental health and motherhood were the most addressed topics. My review of the literature since that time suggests the subfield has not produced much greater breadth. Our findings suggest that gaining a sociopolitical awareness of one’s rights by interrupting traditional gender ideology is a critical and fundamental condition for women’s political participation. In doing so, these findings suggest that a research agenda that prioritizes intersectionality should recognize that inequality and oppression play a role in intersectional experiences and employ analyses of the roles of multiple, simultaneous power injustices in women’s lives.

### Is There a Common Ground for Individuals Who Experience Different Identity Configurations and Oppression? Relatedly, What Are the Mechanisms Required to Become Aware of and Able to Articulate an Intersectional Experience?

As the editors of this Special Issue note, social justice movements, most famously the civil rights movement in the 1960s, succeeded because they brought together people from a variety of social backgrounds working together for social change (Azmitia and Mansfield, this issue). Relatedly, the mobilization and collective identity behind transnational feminism is not based on the idea that women have universal experiences; rather it is rooted in a shared criticism of how neoliberal economic policies and governments create structural conditions that limit women’s rights in their respective locations (Moghadam, 2005). In response to violations that have become increasingly exacerbated in this context, the political mobilization and feminist activity that has emerged reflects diverse modes of resistance, operating from different strategic spaces and subject positions within society (e.g., civil society, participation in social movements, academia) to address women’s growing concerns globally (Ferree and Tripp, 2006; Montenegro et al., 2012). Thus, while it is important not to ignore the unique experiences of women, it is also useful to consider the common experiences shared by marginalized individuals (Azmitia and Mansfield, this issue). In this section I will discuss a methodological approach I used to investigate the mechanisms that emerge when women employ intersectional experiences toward change. Because this investigation was conducted in partnership with women’s social movement actors in Nicaragua, I will describe the social context of the movement before reviewing findings and what we can learn from them.

#### The Women’s Social Movement in Nicaragua

In Nicaragua, women’s experience of gendered injustice has been influenced not simply by the dominant political culture within Nicaragua, but also by decades of United States military intervention and economic policies and practices that have consistently denied women’s rights – all of which serve as powerful intersections between the local and global. Following a catalytic electoral defeat of the Sandinistas in 1990, feminists in Nicaragua came together in ways not yet seen before, in part because they were mobilizing not just outside of the parameters of patriarchal society, but also outside of the parameters of a growing neoliberal context that reflected the extreme marginalization imposed by global capitalism (Randall, 1994). In mapping a model for a social movement, feminist activists in Nicaragua revealed a strategic design whereby actors from different social locations could meet to enact politically effective means for transforming dominant power relations (for a detailed history of the movement, see Grabe, 2016a). In fact, awareness that this political movement brought together women from different backgrounds to work together toward change led to what activists referred to as a “Diverse but United” strategy whereby women’s personal experiences of marginalization, from their various social locations, were used to influence an inclusive strategy for collective mobilization (Grabe and Dutt, 2015). By 1992, several women’s organizations had mobilized into a network under the umbrella name *Movimiento Autónomo de Mujeres* (the Women’s Autonomous Movement) to represent one of the largest, most diverse, and most autonomous feminist movements in Latin America (Kampwirth, 1996).

To best understand how women’s experiences materialized into action within the *Movimiento*, I designed a study that attempted to privilege the standpoint of the activists working in the *Movimiento* (Maddison and Shaw, 2007). The method centered on the use of *testimonios*, a form of oral history or life story that is an explicitly political narrative describing and resisting oppression (Chase, 2003). *Testimonios* have been widely used with Latin American activists involved in revolutionary movements (e.g., Menchú, 1984; Randall, 1994). This method can bring into focus a greater range of activity, showcasing the often invisible and undocumented activity that takes place within social movements (Maddison and Shaw, 2007).

#### The Oral History Project

One of the aims of critical scholarship is to break through the strangleholds imposed by mainstream research agenda and universalizing theories to elevate the voices of marginalized women in the production of knowledge. Therefore, to identify the key women whose *testimonios* would be collected, I relied on suggestions from the elected leader of the *Movimiento*, Juanita Jiménez, and solidarity activist Carlos Arenas, thus building a key sample of participants in the women’s movement as identified by movement activists themselves. During a field visit to Nicaragua in 2011, I conducted 15 interviews with female political activists who were considered key leaders in Nicaragua. To facilitate rapport and ease, the interviews were scheduled in a location of the woman’s choosing. Sometimes this was her home; sometimes it was her office. All of the interviews were preceded by a conversation that involved disclosure of my prior research interests and involvement in Nicaragua as well as my political commitments and motivations for this project; and each woman was given copies (in Spanish) of the research I had conducted in the past. Before beginning the interviews, I explained to each woman how her story might be used and disseminated [i.e., through the Global Feminisms Project, (GFP)^7^ a book, journal manuscripts] and each was given a list of the other interviewees in order to know whom the other participants would be. All of the women agreed enthusiastically to participate in the project and have her story reproduced. The women I interviewed were former guerrilla commanders, occupied ministerial or congressional positions, were heads of human rights counsels, journalists, grassroots organizers, academics, labor union organizers, Nobel Peace Prize nominees, and rural feminists. Nine of their *testimonios* were included in the book *Narrating a Psychology of Resistance: Voices of the Compañeras in Nicaragua* (Grabe, 2016a), published by Oxford University Press. Eleven of the interviews, in their entirety, can be viewed and downloaded in English and Spanish from the GFP website.

To demonstrate how understanding and rejecting social obstacles to actualizing women’s rights formed a “common ground” for women to articulate their experiences, I will reproduce here portions of the *Narrating a Psychology of Resistance* book. Section two of the book focused specifically on intersectional ideology. In moving ideology to action, all of the women in that section of the book (Violeta Delgado, Sandra Ramos, and Bertha Inès Cabrales) invoked processes related to *conscientización*. Brazilian social theorist Freire (1970) concept of *conscientización* refers to a process in which those working to create bottom-up social change participate in an iterative, ideological process whereby analysis and action develop together in a limited situation. The *testimonios* of Violeta, Sandra, and Bertha underscore how an oppositional ideology was used to build laws that responded to women’s situations. Violeta’s story helped illustrate that an oppositional ideology that viewed women’s experiences as valid sources of knowledge could facilitate an understanding of violations against women as systemic and in need of transformation via legislation that upholds women’s right to live with dignity. Violeta’s efforts underscored how a transnationally intersectional perspective influenced awareness and action because her oppositional ideology to the treatment of women in Nicaragua led to her to strategically use international discourse and alliance to build awareness and put pressure on the Nicaraguan National Assembly to enact laws to protect women from violence. Sandra’s *testimonio* highlighted how *conscientización* is an iterative process of learning that enhances one’s contribution as a citizen subject by facilitating others’ positions as subjects who know and can claim their rights within a transnational system that marginalizes their experience. Bertha’s *testimonio* underscored that oppositional ideology needs to come from the margins in order to create change by rejecting a “symbolic” form of politics in favor of legislation that questions abuses of power and serves to protect the interests of women. All three women used their oppositional ideology to find “common ground” by opposing abuses of power and trying to restore them through legal action related to women’s rights – creating laws, using human rights discourse to monitor factories, or educating the masses in feminist theory that explained how gender subordination puts women at risk for rights violations. Taken together, the *testimonios* of these women suggest that to be effectively positioned to enhance women’s agency as subjects with rights it was necessary to employ an intersectional ideology that opposed a social order whereby men retain power and use it within a capitalist system that, by design, violates women’s rights. The findings from this particular investigation suggest that a research agenda rooted in investigating the structural dimensions of power should prioritize how intersectional experiences can be a vital means for initiating action and creating social change.

### Methodology: Privileging Intersectional Perspectives

Elsewhere others have written in detail about the limits and merits of qualitative versus quantitative approaches in the study of intersectionality (McCall, 2005; Bowleg, 2008; Shields, 2008). I will not propose that adopting intersectionality as a primary analytic tool requires choosing between or among methods. Fine (2012) suggests that in an increasingly neoliberalized context in social science, questions of method and what counts as evidence have contributed to the narrowing of investigations related to matters of justice for women. Fine (2012) states that, “dominant methodologies systematically strip women (and men) of the material and political contexts of their lives; randomly assigning them to condition and/or assessing their outcomes on standardized indicators deemed appropriate by ‘experts’ (p. 10).” The result of this narrowing is that social structures and systems of power (e.g., patriarchy, neoliberalism) related to women’s experiences cannot easily be considered through the use of standard methods. Already within the discussion of intersectionality in psychology there has been ripe debate about *how* to reframe standard methods, with some advocating for the acceptance and use of quantitative methods (e.g., Bowleg and Bauer, 2016; Else-Quest and Hyde, 2016) and others noting the importance of qualitative methods in capturing meaning (Marecek, 2016).

Therefore, rather than advocating one methodological approach over the other, I urge researchers to recognize that there is more than one route to intersectional analyses, that any one study or method offers only what is revealed in that snapshot. Moreover, although I do not believe intersectional analysis mandates a qualitative methodology, I do subscribe to Moradi and Grzanka (2017) call that it *does* mandate the kind of critical self-reflexivity that is common in some qualitative research. The importance in establishing research questions and study design is not in the chosen method, but rather in maintaining an understanding of how intersectional approaches can facilitate the understanding of systems of power, including those reflected in research, regardless of methodological options. Importantly, researchers need to consider how to capture the nuances of how multiple sociohistorical systems and structures of inequality may be impacting women’s identity and experience (Harris and Patton, 2019). Thus, rather than advocating for quantitative versus qualitative, or reproducing debates about their respective limitations, my proposal for researchers is to seek multi method training in order to be positioned to choose the methodological approach that is best suited to the question.

The review of findings provided in this article illustrated the potential benefit to having multiple methodological approaches from which to choose in order to address a number of elements important in intersectional research. First, when considering majority world women’s perspectives in the examination of issues related to social identity categories involving gender, this program of research was able to uncover robust processes related to women’s power and status and human rights. As Elizabeth Cole noted over a decade ago (2009), “considering groups that have traditionally been overlooked may lead researchers to hypothesize about different predictors” (p. 173). Therefore, to me, the challenges in intersectional research lie not so much in the methodology (qualitative versus quantitative), but on the research questions and samples. Although the discipline of psychology, in general, must confront its bias toward the inclusion of participants who are unrepresentative of the majority of the world’s population, this call is even more imperative for intersectional researchers (Nielsen et al., 2017). Despite calls to abandon the habitual dependence on convenience sampling to move toward drawing from diverse samples, the overreliance on WEIRD samples places parameters on many psychologists (Henrich et al., 2010; Cortina et al., 2012). Specifically, the standard employment of college students in social psychology imposes boundaries on the identity categories that can be examined. At a minimum, an intersectional framework requires that participants from multiple marginalized groups be included in research so that their voices are heard (see Else-Quest and Hyde, 2016 for suggested sampling techniques). As such, I implore researchers to consider working with “real world” people, and still more, perhaps with community leaders who can help flesh out understandings that are relevant to the initial research questions.

Secondly, the findings reviewed from the program of research highlighted in this article demonstrated how both quantitative and qualitative analyses were positioned to prioritize the examination of the roles of inequality and oppression in intersectional experiences. Each method allowed for analyses of the roles of multiple, simultaneous power injustices in women’s lives. For example, in the research reviewed in this article, using large scale surveys allowed for the examination of robust psychosocial processes related to inequity and how those social processes may be mediated. In particular, the results suggested that experiences related to women’s human rights – such as violence or political participation – were predicted by factors related to inequity such as gender ideology or relationship power. Perhaps more importantly, the demonstrated processes also illustrated ways in which sociocultural structures of male power could be interrupted to create change. The survey studies, however, did not allow us to deeply capture social context or nuance. Therefore, to better understand the actual lived experience of women, we also used qualitative interviews. These interviews both helped to better understand how the processes operated and to get a better handle on the causal or reciprocal nature of the issues under examination. It was also the case that the survey methods described in this review could not have been used to ask and answer questions related to “common ground.” In this case we used an oral history method, but any qualitative method could have been employed in this case to get at that underlying research focus. The take-home point for me is that an agenda for intersectional research should not privilege a specific methodological approach, but rather it underscores the importance of getting multi method training so that: (1) researchers are well versed enough to choose the right method for the question, rather than needing to design a study based on their training and/or, (2) researchers may employ multiple methods on the same project.

## Conclusion

In this article, I used a decade’s worth of investigations conducted in partnership with women from the majority world to provide examples of methodological approaches that could inform a research agenda on intersectionality – or a framework for investigating multiple sources of oppression in women’s lives. Continued research in psychology can shed light on the diverse experience and development of individuals with intersecting identities and contribute to understanding the role of psychological processes in more effectively challenging the broader structures of power that sustain inequalities. A research agenda rooted in intersectional understandings will require that: (1) researchers think carefully about social categories of analysis and how their methodological choices can best answer those questions, (2) psychologists reposition their research questions to examine processes by which structural inequities lead to power imbalances and norms that sustain individuals’ experiences of marginalization and oppression, and (3) we understand how intersectional experiences can be applied toward change. Because intersectional investigations hold a key to interrupting the structural dimensions of power that result in egregious consequences to peoples’ social, economic, and political lives, it is imperative that we radically restructure what we think about knowledge, our roles, and the products of our research.

## Data Availability Statement

No datasets were generated or analyzed for this study.

## Author Contributions

The author confirms being the sole contributor of this work and has approved it for publication.

## Conflict of Interest

The author declares that the research was conducted in the absence of any commercial or financial relationships that could be construed as a potential conflict of interest. The handling editor declared a shared affiliation, though no other collaboration, with the author SG.

## Footnotes

1 Given that the terms “developing” and “third world” are often used by so called “first world” nations to describe another country in a manner that implies inferiority, the term majority world, borrowed from Kagitcibasi (2002) will be used because individuals from “developing” countries constitute the majority of the world’s population.

2 Despite cautioned use of the term “third world,” women writing from the perspective of the majority world highlight the need for analyses of women’s lives in a manner that reclaims use of “third world” (Mohanty, 1984).

3 Because systems of oppression involving multiple institutions (e.g., patriarchy, capitalism) continue to aggressively limit the situations in which women live their lives, efforts toward liberatory change cannot be understood outside of this context. However, landownership is, of course, embedded within an acceptance of a capitalist model of individual property. Nevertheless, lack of female property ownership in both countries reflected how extreme marginalization imposed by global capitalism becomes gendered. As such, women in both countries were mobilized to facilitate titling women to land they lived on or used.

4 The group of women in Nicaragua was organized through the Xochilt Acalt Women’s Center located in the municipality of Malpaisillo in the state of León, Nicaragua. The organization began shortly after a new presidential administration in 1990 introduced severe structural adjustment that resulted in cutbacks to public sector resources that infringed upon women’s rights. Xochilt Acalt started as a mobilized group of women in 1991 to address high levels of cervical cancer in the remote rural area in which they lived. In subsequent years, Xochilt Acalt formed a community of women (and men) to tackle additional problems and demands that were arising within the community, which included illiteracy, high levels of gender-based violence, lack of resources for family planning, lack of food, high rates of male migration for work, and a need to improve unequal power relations between the genders. In response to the demands, Xochilt Acalt designed and offers workshops in the areas of adult literacy, sexual and reproductive health, small live-stock and veterinary skills, small-scale agricultural production, income-generating programs, and civic participation workshops that advocate for women’s role in democracy and community decision making. A board of eight women directors oversee the overall operation of Xochilt Acalt; however, much of the daily operation is carried out by members based on their experiences, skills, and the needs of the community.

5 MWEDO, in Tanzania, was started in 2000 by three Maasai women interested in promoting gender equality in Maasai communities while at the same time protecting Maasai culture in the face of radical changes happening in the region (Hodgson, 2011). MWEDO implements programs in three primary focus areas: human rights advocacy, women’s economic empowerment, and cultural citizenship that are meant to address the needs raised by members in local communities (e.g., hunger, poverty, lack of clean available water, lack of health care; Hodgson, 2011). Similar to Xochilt Acalt, the programs have in common promotion of women’s rights and advocacy through the use of international human rights frameworks, with the focus of concerns generated by local women (e.g., cultural citizenship) over concerns favored by the international community (e.g., genital cutting; Hodgson, 2011). The programs are administered by MWEDO staff who are employed in a central office, though the membership base includes more than 35 village-based groups spread throughout several local districts (Hodgson, 2011).

6 Given the limitations of cross-sectional data, we also ran alternative path models and considered the qualitative findings before establishing confidence in the causal role of landowning in these processes. The causal direction found in the hypothesized model was supported by the quantitative and qualitative data.

7 The Global Feminisms Project (GFP) is a collaborative international project, housed at the University of Michigan, that conducts, examines, and archives interviews with women involved in feminist activism, social movements, and women’s studies departments in various countries. Currently, the archive consists of interviews with women from Brazil, China, India, Nicaragua, Poland, and the United States. Complete transcripts can be read at https://globalfeminisms.umich.edu/. The GFP project was designed with scholarly goals at its core, therefore with permission from the GFP the interviews are available for future research and teaching across disciplines.
